# The usefulness of "reversed U-curve" HD grid mapping

**DOI:** 10.1016/j.ipej.2024.04.011

**Published:** 2024-05-03

**Authors:** Tatsuya Hayashi, Shingo Yamamoto, Jumpei Ohashi, Hideo Fujita

**Affiliations:** Division of Cardiovascular Medicine, Saitama Medical Center, Jichi Medical University, Saitama, Japan

**Keywords:** Premature ventricular contraction, HD grid, Reversed U-Curve

In a 50-year-old female with frequent Premature Ventricular Contractions (PVCs) displaying a downward-axis left bundle branch block morphology, catheter ablation was performed using the HD Grid in the right ventricular outflow tract (RVOT). At first, the HD Grid was advanced into the RVOT through the tricuspid valve by initially bending it downward, and then by bending it in the opposite direction, following the conventional approach (Fig. 1A). However, the optimal mapping site was slightly lower in the RVOT, and the flexible tip of the HD Grid caused the catheter to slip from the lower RVOT to the RV, resulting in catheter-induced PVCs and impeding effective mapping.

**Fig. 1:**
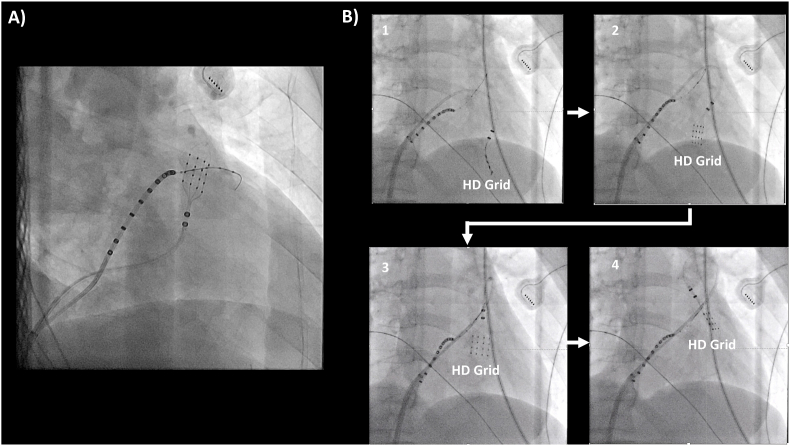
A The figure demonstrates the “conventional approach” to mapping RVOT with the HD Grid catheter (another patient). A wire-type coronary sinus catheter (EPstar Fix AIV and EPstar Fix CS Lumen; Japan Lifeline, Tokyo, Japan) was used to precisely map the outflow tract area. RVOT: right ventricular outflow tract. B The HD grid was bent to its maximum at the right ventricular inflow and delivered to the RVOT with a slight rotation while pushing forward, allowing for the "reversed U-curve technique”.

To overcome this challenge, we employed the reversed U-curve technique. The HD Grid was maximally bent at the RV inflow and inserted directly into the RVOT while being rotated clockwise (Fig. 1B, Supplemental Figure). This technique ensured stable catheter placement in the lower RVOT, allowing for the successful recording of the earliest activation site within the HD Grid (Fig. 2A). Ablation at this site eliminated frequent PVCs (Fig. 2B).

**Fig. 2:**
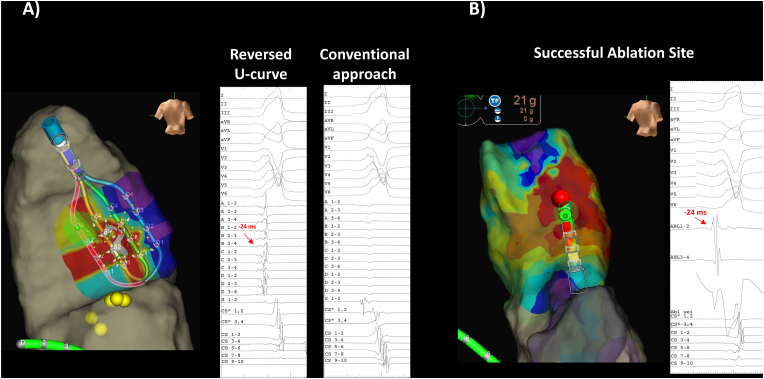
A The reversed U-curve mapping technique identified the earliest activation site of the PVC within the HD Grid, occurring 24 msec earlier than the PVC. Prior mapping using the conventional approach, before employing the reversed U-curve technique, revealed dull potentials, indicating inadequate contact of the HD Grid in the right ventricular outflow tract (RVOT). Only the shaft part of the HD Grid (S1-2) exhibited near-field potential. PVC: Premature Ventricular Tachycardia, CS: coronary sinus (A–D represents each spline of the HD grid, CS” represents the distal site of the coronary sinus). B The successful ablation site and its local potential is exhibited.

The HD Grid, known for its high-quality mapping capabilities [1], typically minimizes catheter-induced PVCs due to its electrode configuration. However, when mapping the RVOT with the HD Grid catheter, the earliest activation site often lies slightly lower in the RVOT, presenting challenges in catheter stability. To address these problems, we demonstrate for the first time the effectiveness of the reversed U-curve technique. The reversed U-shape technique allows the “moving point” of the HD Grid catheter to be placed above the fan-shaped electrode tip, which is thought to provide more stable mapping in the RVOT. Because the left and right outflow tracts have an elongated cylindrical shape, and the HD Grid is flat, achieving complete contact between the HD Grid and the ventricular surface can be problematic. In addition to the conventional approach, the reversed U-curve technique improved the contact area between the HD grid and RVOT, resulting in better mapping. Furthermore, recent studies have shown the usefulness of recording outflow tract potentials directly from above the pulmonary valve [2], and the present reversed U-curve mapping demonstrates the possibility of doing it with a multielectrode catheter.

The reversed U-curve technique is a useful mapping technique that extends the capabilities of the HD grid and overcomes its limitations.

## Disclosures

No conflicts of interest related to this topic.

## Ethics approval statement

Accepted (臨S23-190).

## Patient consent statement

Informed consent obtained.

## Clinical trial registration

N/A.

## Ethical statement

This case report was approved by the institutional review board of Saitama Medical Center, Jichi Medical University (S23-190).

## Declaration of competing interest

The authors declare that they have no known competing financial interests or personal relationships that could have appeared to influence the work reported in this paper.
